# The intratracheal administration of locked nucleic acid containing antisense oligonucleotides induced gene silencing and an immune-stimulatory effect in the murine lung

**DOI:** 10.1371/journal.pone.0187286

**Published:** 2017-11-06

**Authors:** Yasunori Uemura, Kenji Hagiwara, Katsuya Kobayashi

**Affiliations:** 1 Immunology & Allergy R&D Unit, R&D Division, Kyowa Hakko Kirin Co., Ltd., Nagaizumi-cho, Shizuoka, Japan; 2 Innovative Technology Labs, Research Functions Unit, R&D Division, Kyowa Hakko Kirin Co., Ltd., Machida, Tokyo, Japan; Medical University of South Carolina, UNITED STATES

## Abstract

Locked nucleic acid containing antisense oligonucleotides (LNA-ASOs) have the potential to modulate the disease-related gene expression by the RNaseH-dependent degradation of mRNAs. Pulmonary drug delivery has been widely used for the treatment of lung disease. Thus, the inhalation of LNA-ASOs is expected to be an efficient therapy that can be applied to several types of lung disease. Because the lung has a distinct immune system against pathogens, the immune-stimulatory effect of LNA-ASOs should be considered for the development of novel inhaled LNA-ASOs therapies. However, there have been no reports on the relationship between knock-down (KD) and the immune-stimulatory effects of inhaled LNA-ASOs in the lung. In this report, LNA-ASOs targeting Scarb1 (Scarb1-ASOs) or negative control LNA-ASOs targeting ApoB (ApoB-ASOs) were intratracheally administered to mice to investigate the KD of the gene expression and the immune-stimulatory effects in the lung. We confirmed that the intratracheal administration of Scarb1-ASOs exerted a KD effect in the lung without a drug delivery system. On the other hand, both Scarb1-ASOs and ApoB-ASOs induced neutrophilic infiltration in the alveoli and increased the expression levels of G-CSF and CXCL1 in the lung. The dose required for KD was the same as the dose that induced the neutrophilic immune response. In addition, in our in vitro experiments, Scarb1-ASOs did not increase the G-CSF or CXCL1 expression in primary lung cells, even though Scarb1-ASOs exerted a strong KD effect. Hence, we hypothesize that inhaled LNA-ASOs have the potential to exert a KD effect in the lung, but that they may be associated with a risk of immune stimulation. Further studies about the mechanism underlying the immune-stimulatory effect of LNA-ASOs is necessary for the development of novel inhaled LNA-ASO therapies.

## Introduction

Nucleic acid therapy has potential to be a next-generation therapy because it can modulate molecules that cannot generally be targeted using small molecules or antibodies. Antisense oligonucleotides (ASOs) are synthetic single-stranded strings of nucleic acids that bind to RNA through standard Watson-Crick base pairing [1]. In particular, locked nucleic acid containing antisense oligonucleotides (LNA-ASOs) possess an extremely high binding affinity to complementary RNA oligonucleotides, display improved mismatch discrimination and shows high stability in biological systems in comparison to conventional ASOs without any chemical modifications of their gap positions [2–4]. Several studies have indicated that LNA-ASOs exert not only a knock-down (KD) effect on cell lines without the use of transfection reagents such as lipofectamine [5–7], but that they also exert a KD effect in vivo, without any drug delivery system [8–12].

The systemic delivery of ASOs has been performed by intravenous or subcutaneous injection, and broad accumulation was observed in various tissues. This “undesired” accumulation would increase the risk of inducing side effects in organs that are not the target of treatment [13]. Pulmonary drug delivery has the potential to minimize systemic exposure and decrease the risk of adverse effects of LNA-ASOs. This direct and non-invasive delivery system has been widely used for the treatment of lung disease [14–17]. In addition, saline-based solutions are a suitable carrier for ASOs. These compounds have been shown to be able to withstand the nebulization process [18, 19]. Thus, inhalation therapy with LNA-ASOs represent a potential next-generation therapy for numerous respiratory diseases.

The lung has a distinct immune system that protects against pathogens [20, 21]. Alveolar macrophages, which are the resident phagocytes in the lung, reside in the air space. They play a pivotal role in the host defense and in the lung immune response [22, 23]. Epithelial cells on the lung surface also strongly contribute to the pulmonary immune response [24, 25]. In terms of the structure of the lung and the distribution of these two types of cells, these are the first cells that inhaled LNA-ASOs contact. Thus, for the development of novel inhaled LNA-ASOs therapies, it is necessary to evaluate the immune-stimulatory effect of LNA-ASOs. However, there have been no reports that clearly show the relationship between KD and the immune-stimulatory effect of inhaled LNA-ASOs.

Scavenger receptor BI (Scarb1) is a high-density lipoprotein receptor expressed in several tissues, including the lung. In our experiment, the non-normalized expression of Scarb1 in the murine lung was the same as that of house-keeping gene Hprt1. And the systemic administration of LNA-ASOs targeting this gene has been shown to exert a KD effect in mouse lung [26, 27]. We therefore selected Scarb1 as the target gene to evaluate the KD effect. For negative control LNA-ASOs, we prepared LNA-ASOs targeting apolipoprotein B (ApoB). ApoB is an essential protein for the assembly and secretion of very-low-density and low-density lipoprotein and is expressed predominantly in the liver and jejunum [28]. Regarding the immune response, Scarb1-null mice showed a higher sensitivity to LPS than wild type mouse, but the deletion of Scarb1 did not directly induce an abnormal immune response [29, 30], and there have been no reports of the KD of ApoB directly inducing an immune response in lung. These findings suggest that the immune-stimulatory effect of LNA-ASOs targeting Scarb1 or ApoB can be evaluated without being affected by a KD. These LNA-ASOs exerted a KD effect without transfection reagents in vitro. Then, to investigate KD and the immune-stimulatory effect on lung, these LNA-ASOs were intratracheally administered to mice. In addition, we investigated whether the immune-stimulatory effect of LNA-ASOs in vivo can be estimated by an in vitro assay using primary mouse lung cells.

## Materials and methods

### Mice

Eight-week-old female specific-pathogen-free C57BL/6 mice were purchased from the Charles River Laboratories Japan, Inc. (Ibaraki, Japan), and kept in the Laboratory of Kyowa Hakko Kirin for 1 week prior to the experiments. All animals were maintained at 20–26°C and 30%-70% relative humidity with a 12 h light–dark cycle, and had free access to a standard rodent diet and water. The experiments were performed when the mice were 9–10 weeks of age, weighing 17–22 g. All efforts were made to minimize animal suffering and to use the minimum number of animals necessary to produce reliable scientific data. The experiments were performed when the mice were 9–10 weeks of age. All of the animal studies were performed in accordance with Standards for Proper Conduct of Animal Experiments at Kyowa Hakko Kirin Co., Ltd. under the approval of the company’s Institutional Animal Care and Use Committee. Kyowa Hakko Kirin Co., Ltd. is fully accredited by the Association for the Assessment and Accreditation of Laboratory Animal Care, International.

### Oligonucleotides

Locked nucleic acid antisense oligonucleotides (LNA-ASOs) complementary to the mouse Scarb1 or ApoB mRNA were chemically synthesized by GeneDesign, Inc. (Osaka, Japan).

The sequences of the ASOs were as follows.

Scarb1: LT^LC^A^G^T^C^A^T^G^A^C^T^LT^LC
ApoB: LG^LC^A^T^T^G^G^T^A^T^LT^LC^LA
A, T, G, C: DNA; ^: Phosphorothioate bond; LN: LNA; LC: LNA 5-Methyl cytosine

### Mouse cryopreserved hepatocytes and culture

Mouse (CD-1) Cryopreserved Hepatocytes and culture medium were purchased from Thermo Fisher Scientific (Waltham, MA, USA). Mouse hepatocytes were suspended in plating medium consisting of Williams medium with Hepatocyte Plating Supplement Pack (Serum Containing). Suspended cells were seeded at 1 x 10^4^ /well in a type I collagen-coated 96-well flat bottom plate (Corning, New York City, USA) and treated with 30, 100, 300 and 1000 nmol/L of Scarb1-ASOs or ApoB-ASOs. After 6 h, the culture medium was exchanged for incubation medium consisting of Williams Medium E (no phenol red) with Hepatocyte Maintenance Supplement Pack (Serum Free), and cultured for 18 h.

### The preparation of primary mouse lung cell suspension and culture

Primary mouse lung cell suspension was prepared using a gentleMACS Dissociator (Miltenyi Biotec, Bergisch Gladbach, Germany) with reference to the modified protocol of the Lung Dissociation Kit (Miltenyi Biotec). Briefly, 3 mice were euthanized with CO_2_ in an appropriate chamber and lung was harvested. The lungs were digested by DMEM supplemented with penicillin-streptomycin (Thermo Fisher scientific) and 1.5 mg/mL of collagenase A (Roche Diagnostics, Basel, Switzerland), and then the 37C_m_LDK_1 and m_lung_02 programs of the gentleMACS Dissociator were run. After digestion, the erythrocytes in the lung cell suspension were lysed with BD Pharm Lyse Lysing Solution (BD biosciences, San Jose, CA, USA). Prepared lung cells were seeded at 5 x 10^5^ /well in a 96-well flat bottom plate (Nunc, Roskilde, Denmark) and treated with 8, 40, 200, 800 and 2000 μg/mL of Scarb-1-ASOs or ApoB-ASOs, for 24 h.

### Flow cytometry

Lung cells were suspended in a FACS buffer of PBS with 2 mmol/L EDTA (Thermo Fisher scientific) and 2v/v% FBS (Thermo Fisher scientific) and incubated with a cocktail of monoclonal antibodies, which included PE Rat Anti-Mouse CD11b, Anti-Mouse CD326 (EpCAM) APC (BD biosciences), Anti-Mouse CD45 FITC, Anti-Mouse F4/80 Antigen APC (ebioscience, San Diego, CA, USA), and PE/Cy7 Anti-mouse CD11c Antibody (Biolegend, San Diego, CA, USA). Flow cytometry was performed using a FACS Verse (BD Biosciences) and the FlowJo software program (Treestar). Alveolar macrophages were characterized as CD45+ CD11b- CD11c+ F4/80+, and lung epithelial cells were characterized as CD45- CD326+.

### The intratracheal LNA-ASOs administration

Sixteen mice were randomly assigned to the 4 groups (N = 4). Scarb1-ASOs was dissolved in sterile saline solution and applied by a MicroSprayer MS-IA-1C (Penn-Century, Wyndmoor, PA, USA) as a single dose of 10, 40 or 100 μg in 50 μL per mouse under light anesthesia with 2.5% isoflurane. The control mice received 50 μL of saline. The volume of intratracheal administration was referred to previous report [31]. Scarb1-ASOs was administered once daily on two consecutive days. One day after the last administration, the right lung or bronchoalveolar lavage fluid (BALF) was harvested for the mRNA or protein expression analyses. The experiment with a focus on ApoB-ASOs was performed with same protocol as Scarb1-ASOs.

### The analysis of BALF

One day after the last administration of ASOs, mice were sacrificed by exsanguination under anesthesia with 2.5% isoflurane, then BALF samples were collected by making an incision in the trachea and washing the lungs twice with 0.75 mL PBS (Thermo Fisher Scientific). BALF samples from each mouse were centrifuged at 950 g for 5 min at 4°C. The total cell counts in the cell pellet were determined using a Sysmex KX-21NV (Sysmex, Kobe, Japan), and the cell population was analyzed using Diff-Quick-stained cytospin preparations, as previously described [32, 33]. The supernatant was used for neutrophil-related cytokine/chemokine measurement using the Bio-Plex 200 system (BioRad, Hercules, CA, USA), with the detection limit set at 0.

### The quantitative RT-PCR

After the culturing of mouse hepatocytes, total RNA was extracted and converted to cDNA using SuperPrep Cell Lysis & RT Kit for a qPCR (TOYOBO, Osaka, Japan), according to the manufacturer’s protocol. After culturing the primary mouse lung cells, total RNA was extracted using a Maxwell RSC simplyRNA Cells Kit (Madison, WI, USA) and MaxWell RSC (Promega). The total RNA was converted to cDNA using a SuperScript VILO cDNA Synthesis Kit (Invitrogen), according to the manufacturer’s protocol. The lungs from LNA-ASOs-treated mice were homogenized using a homogenization buffer, Buffer RLT (QIAGEN, Hilden, Germany) with 1 mol/l-Dithiothreitol Solution (Nakarai, Kyoto, Japan), and tissue lyser II (QIAGEN). Total RNA was extracted from the lysate using a Maxwell RSC simplyRNA Tissue Kit (Promega) and MaxWell RSC, and cDNA was synthesized using SuperScript VILO, according to the manufacturer’s protocol. Each mRNA level was evaluated by a quantitative RT-PCR using TaqMan Gene Expression Master Mix or TaqMan Fast Universal PCR Master Mix (Thermo Fisher Scientific), The TaqMan Probes listed below and QuantStudio 12K flex (Thermo Fisher Scientific) were used. The probes were as follows: mouse Scarb1, Mm00450234_m1; mouse ApoB, Mm01545156_m1; mouse Actb, Mm00607939_s1; mouse Hprt1, Mm01545399_ml; mouse G-CSF, Mm00438334_m1; and mouse Cxcl1, Mm04207460_m1. The relative mRNA expression was quantified using the comparative CT Method.

### Statistical analysis

The statistical significance of the KD or immune-stimulatory effect in vitro was calculated by the Williams test or Aspin-Welch test. The statistical significance of the KD effect in vivo was calculated by the Williams test, the in vivo immune responses were analyzed by the Wilcoxon rank sum test using the SAS software program. P values of < 0.05 were considered to indicate statistical significance.

## Results

### The KD effect of LNA-ASOs *in vitro*

**Fig 1:**
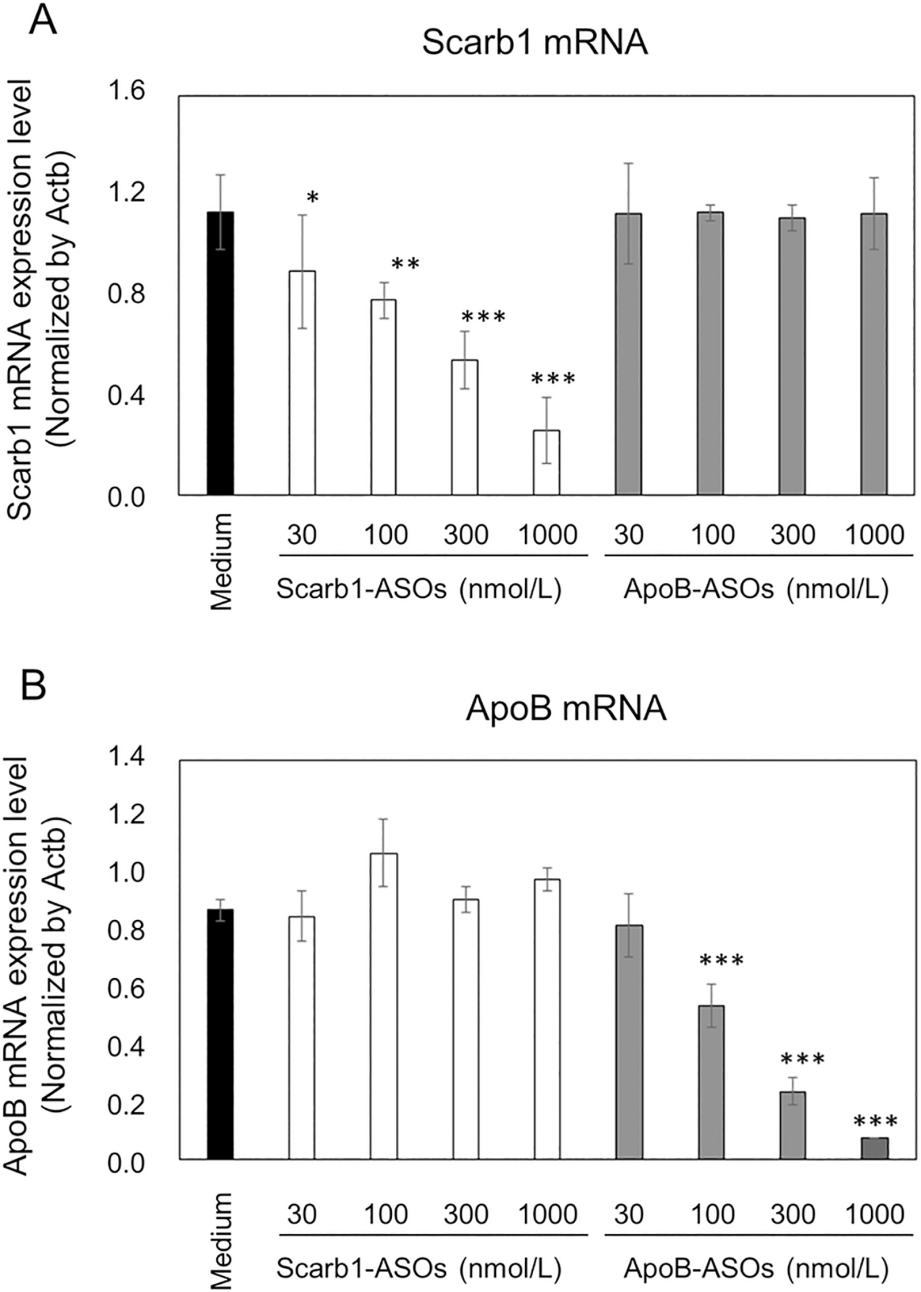
The KD effects of Scarb1-ASOs and ApoB-ASOs on mouse hepatocytes *in vitro*. Mouse hepatocytes were treated with the indicated concentrations of Scarb1-ASOs (A) or ApoB-ASOs (B). After the treatment, the expression levels of Scarb1 or ApoB mRNA were measured. The values represent the mean ± SD of triplicate experiments. ***, P < 0.001, **, P < 0.01, *, P < 0.05 versus Medium group (Williams test).

To determine the KD effect of LNA-ASOs *in vitro*, primary mouse hepatocytes were treated with 30 to 1000 nmol/L of Scarb1-ASOs or ApoB-ASOs. In comparison to the medium-treated cells, Scarb1-ASOs suppressed Scarb1 mRNA with a maximum value of 80% at 1000 nmol/L, but did not affect ApoB mRNA (Fig 1A). In addition, ApoB-ASOs induced a KD effect with maximum value of 90% at 1000 nmol/L, without the suppression of the Scarb1 mRNA expression (Fig 1B). These results indicated that both LNA-ASOs showed the sequence-specific KD of mRNA *in vitro*.

### The KD effect of the intratracheal administration of Scarb1-ASOs in the mouse lung *in vivo*

**Fig 2:**
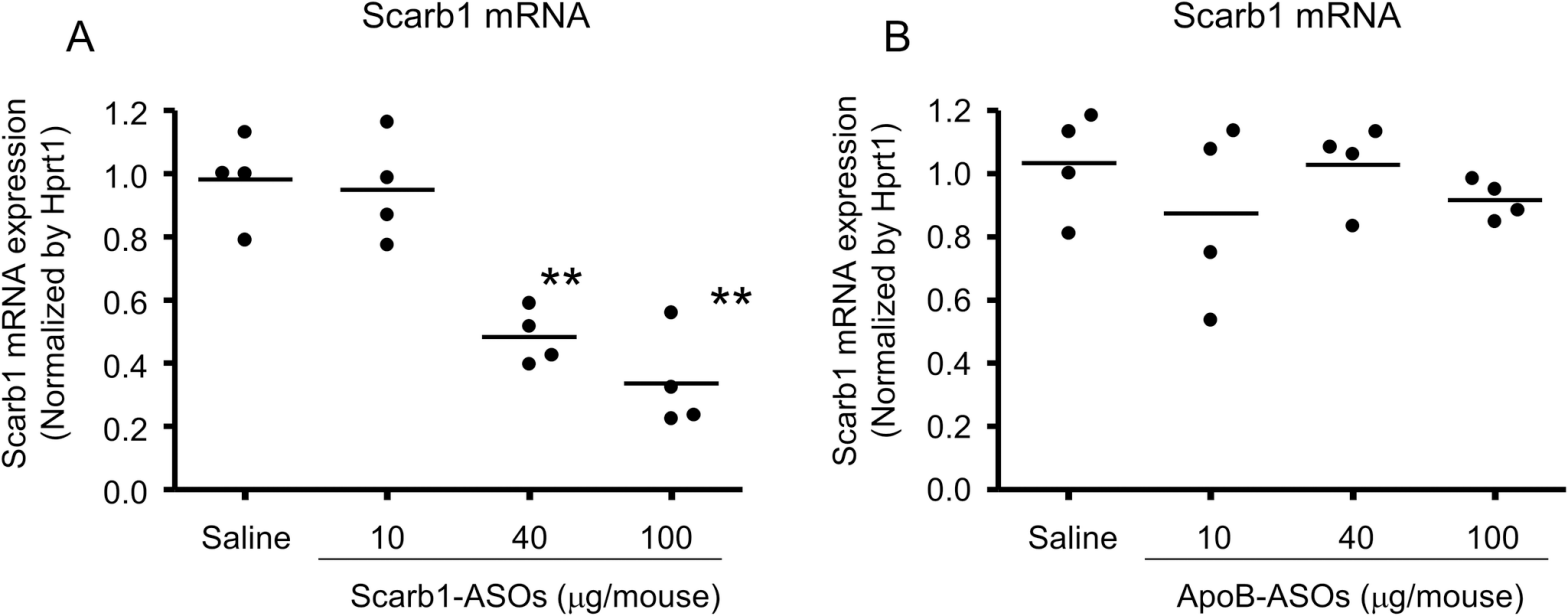
The KD effect of Scarb1-ASOs on the mouse whole lung *in vivo*. Scarb1-ASOs (A) or ApoB-ASOs (B) were intratracheally administered to C57BL/6 mice once a day, for 2 days. One day after the final administration, the right lung was collected and the Scarb1 mRNA expression was measured. The dots indicate each measurement in mice (n = 4). Horizontal bars indicate the mean values. **, P < 0.01 versus the Saline group (Williams test).

Subsequently, the KD effect of Scarb1-ASOs was evaluated in the mouse lung *in vivo*. ApoB-ASOs were used as a negative control. Ten, 40 or 100 μg of LNA-ASOs per mouse were intratracheally administered to mice using a MicroSprayer, once a day for 2 days. One day after the last administration, total mRNA was extracted from the right whole lung, and the Scarb1 mRNA expression level was quantified. In the group treated with 40 μg/mouse or 100 μg/mouse, 50 or 65% suppression was observed, respectively (Fig 2A). This suppression was significant and dose-dependent. On the other hand, the intratracheal administration of ApoB-ASOs did not suppress the Scarb1 mRNA expression in the lung (Fig 2B). These results indicated that Scarb1-ASOs also showed the sequence-specific KD of mRNA *in vivo*.

### The effect of LNA-ASOs on the immune response in the mouse lung

**Fig 3:**
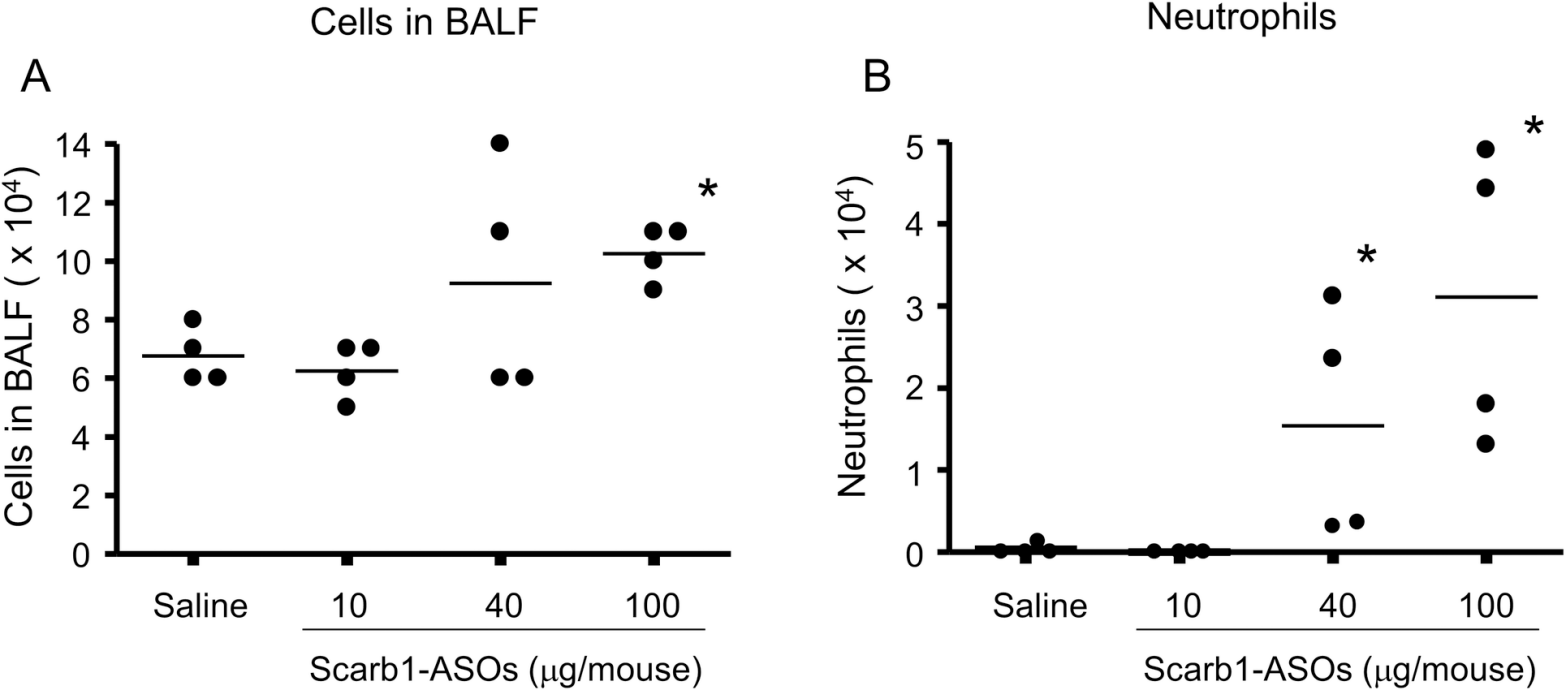
The effect of Scarb1-ASOs on the total cell count and the number of neutrophils in bronchoalveolar lavage fluid (BALF). Scarb1-ASOs were intratracheally administered to C57BL/6 mice once a day, for 2 days. One day after the final administration, BALF was harvested and the total cells (A) and neutrophils (B) in the BALF were analyzed. The dots indicate each measurement in mice (n = 4). Horizontal bars indicate the means. *, P < 0.05 (Wilcoxon rank sum test).

The immune-stimulatory effect of the intratracheal administration of Scarb1-ASOs on the mouse lung was evaluated. After the intratracheal administration of Scarb1-ASOs, the total number of cells and neutrophils in the BALF were counted. Scarb1-ASOs increased both the total number of cells (Fig 3A) and the neutrophils in the BALF, in a dose-dependent manner (Fig 3B). A significant increase in the total number of cells was observed in the group that received Scarb1-ASOs at a dose of 100 μg/mouse. Significant increases were also observed in the number of neutrophils in the groups that received doses of 40 μg/mouse and 100 μg/mouse.

**Fig 4:**
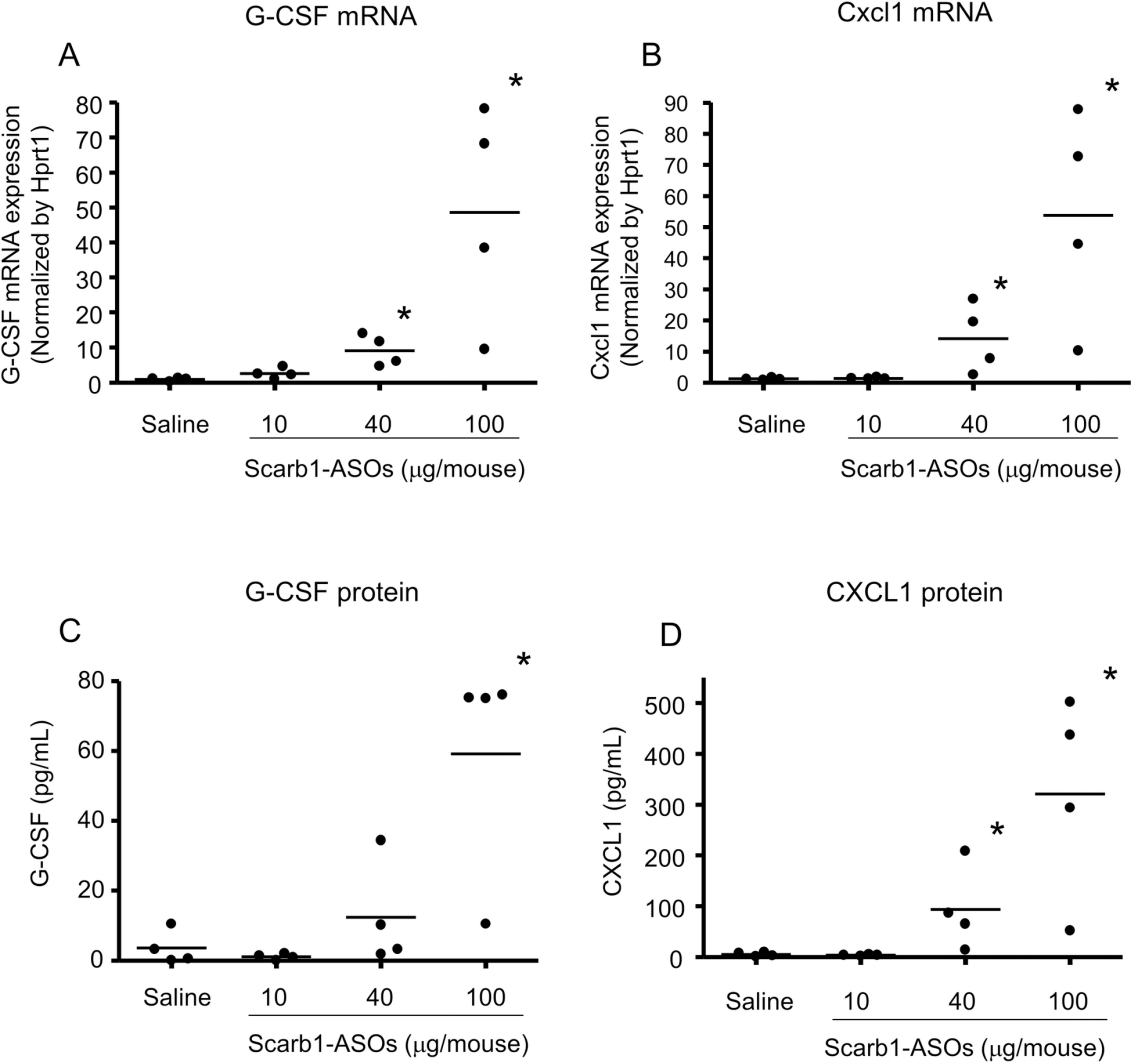
The effect of Scarb1-ASOs on the G-CSF and CXCL1 expression in the lung. Scarb1-ASOs were intratracheally administered to C57BL/6 mice once a day, for 2 days. One day after the final administration, the right lung and BALF were collected and the G-CSF (A) and Cxcl1 mRNA (B) expression in the lung, and the G-CSF protein (C) and CXCL1 protein (D) expression in the BALF were measured. Dots indicate each measurement in mice (n = 4). Horizontal bars indicate the mean value. *, P < 0.05 (Wilcoxon rank sum test).

Based on the observation of neutrophil infiltration in the alveoli, it was hypothesized that neutrophil-related cytokines and chemokines, such as G-CSF [34, 35] and Cxcl1 [36, 37], were induced in the lung. The G-CSF and Cxcl1 mRNA expression was analyzed after the intratracheal administration of Scarb1-ASOs. In comparison to the saline-treated group, the expression levels of both G-CSF and Cxcl1 mRNAs were significantly increased in the groups of mice that received Scarb1-ASOs at doses of 40 μg/mouse or 100 μg/mouse (Fig 4A and 4B). Furthermore, the protein levels of both cytokine and chemokine in the BALF were increased in a dose-dependent manner (Fig 4C and 4D). A significant increase in the G-CSF protein level was observed in the mice that received Scarb1-ASOs at a dose of 100 μg/mouse, while a significant increase in the CXCL1 protein level was observed in the groups that received 40 μg/mouse and 100 μμg/mouse (in comparison to the group that received saline). Moreover, the intratracheal administration of ApoB-ASOs at doses of 40 μg/mouse or 100 μg/mouse significantly induced the expression of these mRNAs and proteins in the lung in comparison to the group that received saline (S1 and S2 Figs). Regarding other inflammatory cytokines, a significant increase in the IL-6 protein/mRNA was observed in the groups that received 100 μg/mouse of Scarb1 or ApoB-ASOs (data not shown).

### The effect of LNA-ASOs on primary lung cells *in vitro*

**Fig 5:**
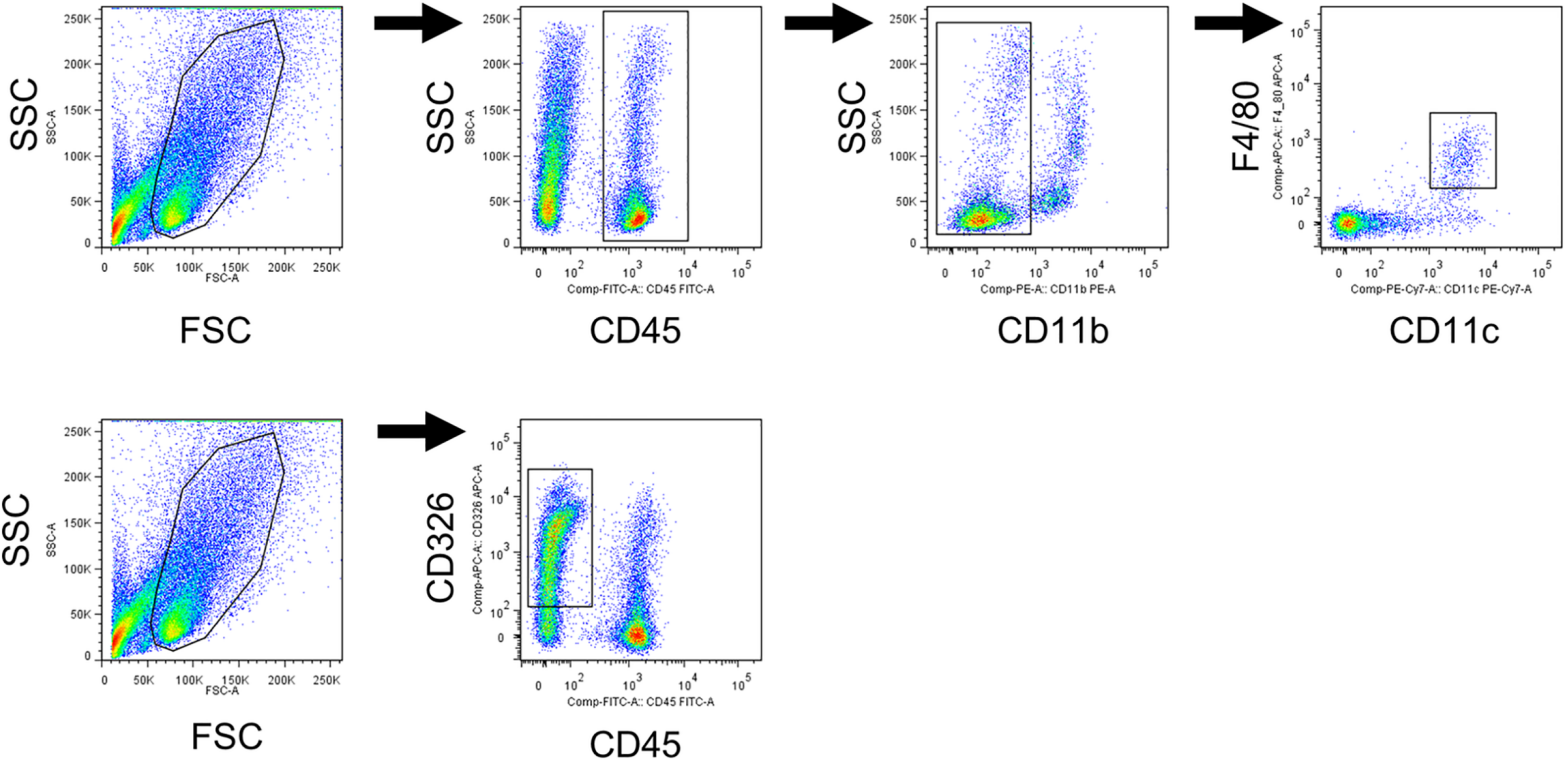
The expression of cell surface markers in primary lung cell suspension derived from the mouse lung. The suspensions were harvested and alveolar macrophages and lung epithelial cells were identified using flow cytometry. Alveolar macrophages were identified as CD45+, CD11b-, F4/80+, and CD11c+. The lung epithelial cells were identified as CD45-, CD326+.

**Fig 6:**
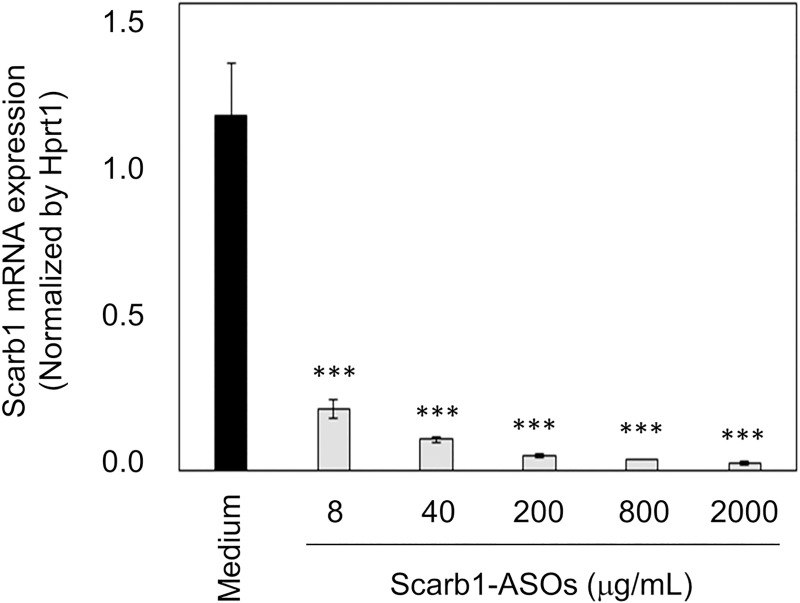
The KD effect of Scarb1-ASOs on primary lung cells *in vitro*. Primary lung cells were treated with the indicated concentrations of Scarb1-ASOs for 24 h. The Scarb1 mRNA expression was measured after the treatment. The left Y axis shows the values for the Scarb1-ASO or medium-treated cells. The right Y axis shows the value for the R848-treated cells. The values represent the mean ± SD of triplicate experiments. ***, P < 0.001, versus Medium group (Williams test).

**Fig 7:**
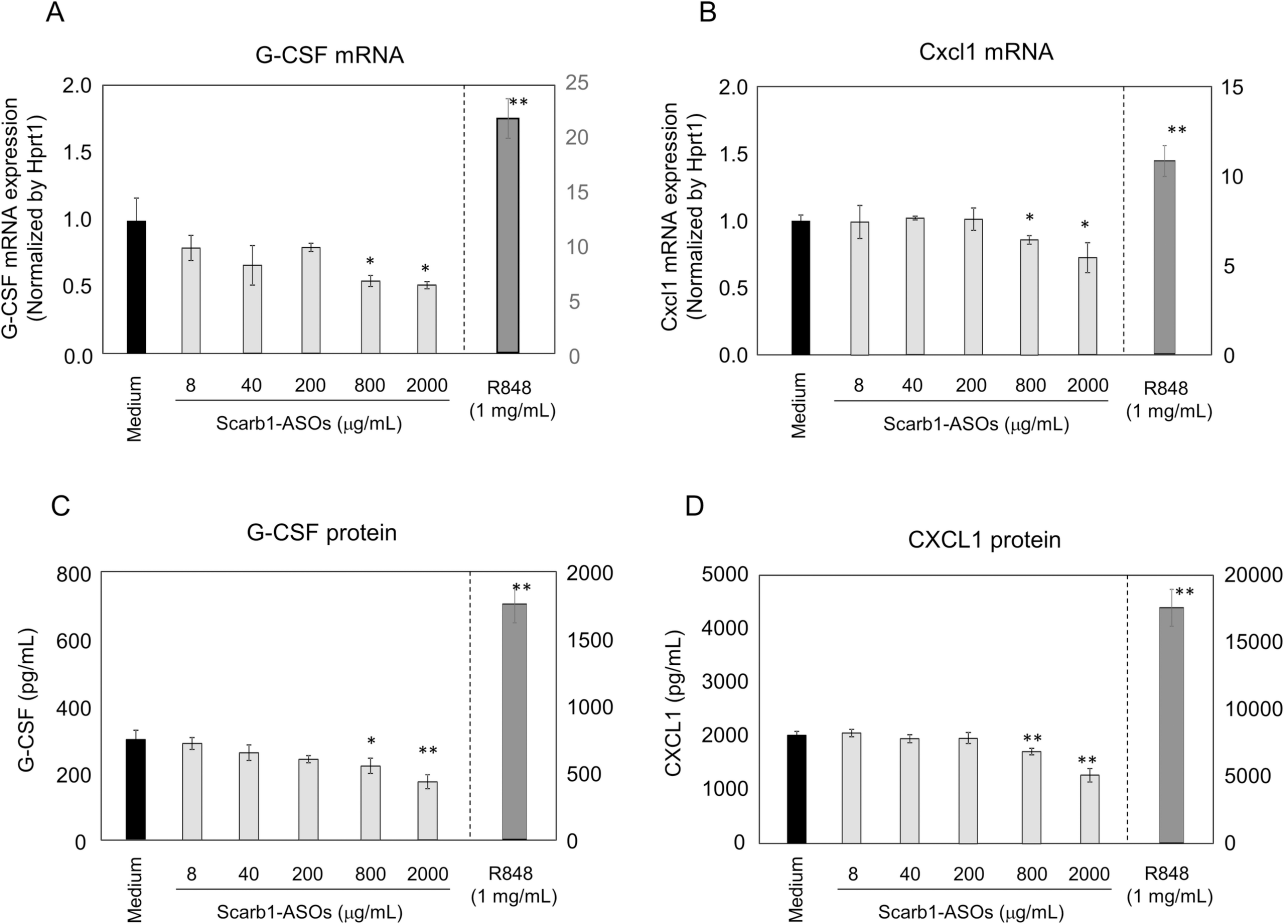
The effect of Scarb1-ASOs on the G-CSF and CXCL1 expression of primary lung cells. Primary lung cells were treated with the indicated concentrations of Scarb1-ASOs or R848 for 24 h. After the treatment, the G-CSF (A) and CXCL1 (B) mRNA expression in primary lung cells, and the G-CSF (C) and CXCL1 (D) protein expression in the supernatant were measured. The left Y axis shows the value for the Scarb1-ASO- or medium-treated cells. The right Y axis shows the value for the R848-treated cells. The values represent the mean ± SD of triplicate experiments. **, P < 0.01, *, P < 0.05 versus Medium group (Aspin-Welch test).

It was hypothesized that LNA-ASOs stimulated cells would have the potential to produce G-CSF and Cxcl1 in the lung. To investigate this hypothesis, a primary lung cell suspension was prepared from normal mouse lung and was treated with 8 to 2000 μg/mL of Scarb1-ASOs *in vitro*. Flow cytometry confirmed that this lung cell suspension contained alveolar macrophages and lung epithelial cells (Fig 5). In terms of the KD effect of Scarb1-ASOs on lung cells *in vitro*, 8 to 2000 μg/mL of Scarb1-ASOs strongly suppressed the expression of Scarb1 mRNA by 85 to 95% in comparison to the medium-treated cells (Fig 6). In this experiment, 1 μg/mL of R848, TLR7/8 agonist [38], was used as a positive control for the evaluation of immune stimulation. In the R848-treated cells, the expression levels of both G-CSF and Cxcl1 mRNA were increased to levels that were 10 to 20 times higher than those of medium-treated cells (Fig 7A and 7B). On the other hand, any dose of Scarb1-ASOs did not increase the expression of G-CSF or Cxcl1 mRNA (Fig 7A and 7B). We also measured the G-CSF and CXCL1 protein expression in supernatant. R848 increased the production of both G-CSF and CXCL1 proteins, whereas Scarb1-ASOs did not increase the production at all (Fig 7C and 7D). These results were clearly correlated with the results of the mRNA analysis. Next, we carried out the same *in vitro* experiment using ApoB-ASOs. ApoB-ASOs did not increase the expression of G-CSF or CXCL1 (S3A–S3D Fig).

## Discussion

In this report, we investigated KD and the immune-stimulatory effect of the intratracheal administration of LNA-ASOs on the mouse lung *in vivo*. As experimental tools, two types of LNA-ASOs targeting Scarb1 or ApoB mRNA were prepared. *In vitro*, these LNA-ASOs exerted a robust KD effect on primary mouse hepatocytes without the use of a transfection reagent (Fig 1A and 1B). In this assay, ApoB-ASOs did not affect the expression of Scarb1 mRNA. To investigate the KD effect in the lung *in vivo*, these LNA-ASOs were intratracheally administered to mice. We confirmed that intratracheal administration of Scarb1-ASOs significantly suppressed the expression of Scarb1 mRNA in the lung in a dose-dependent manner (Fig 2A). On the other hand, ApoB-ASOs did not reduce the expression of Scarb1 mRNA (Fig 2B). These results indicated that the intratracheal administration of Scarb1-ASOs exerted a sequence-dependent KD effect in the lung *in vivo*, without DDS.

Because the lung is always faced with airborne pathogens, it has a distinct immune system against pathogens [20, 21]. Actually, the intratracheal administration of toll like receptor agonists (such as CpG dinucleotides) induces innate immune responses in the lung via pattern recognition receptors [39]. Thus, inhaled LNA-ASOs may be associated with a risk of inducing such responses in the lung. In this report, we confirmed that the intratracheal administration of Scarb1-ASOs increased the expression levels of G-CSF and CXCL1 in the lung and induced neutrophilic infiltration in the alveoli (Figs 3 and 4). The dose that induced a neutrophilic immune response was the same as the dose that was required for KD *in vivo*. The intratracheal administration of ApoB-ASOs also induced similar immune responses in the lung without the suppression of the Scarb1 mRNA expression (S1 and S2 Figs). These results suggested that the neutrophilic immune response was not caused by Scarb1 mRNA KD and that inhaled LNA-ASOs, even without the CpG motif, are probably associated with the risk of inducing a neutrophilic immune response. These results also suggested that we should consider the immune-stimulatory effect of LNA-ASOs when evaluating the drug effect of intratracheally administered LNA-ASOs targeting disease-associated genes in an animal model reflecting human disease, otherwise the results may be misunderstood.

We subsequently investigated whether the immune-stimulatory effect of LNA-ASOs *in vivo* could be estimated by an *in vitro* assay. For this investigation, primary lung cells were prepared and treated with Scarb1-ASOs *in vitro*. This lung cell suspension contained alveolar macrophages and lung epithelial cells (Fig 5). These cells have potential to induce inflammatory responses [21–24], and—in comparison to other lung cells—would be exposed to higher concentrations of LNA-ASOs after intratracheal administration. Actually, the treatment of R848 increased the G-CSF and CXCL1 expression in prepared lung cells *in vitro* (Fig 7A–7D). These results suggested that prepared primary lung cells contained appropriate cells for investigating the immune-stimulatory effect observed in our *in vivo* experiment. Because the intratracheal administration of 100 μg of LNA-ASOs in 50 μL saline solution (2000 μg/mL) induced a neutrophilic immune response in the lung, it was hypothesized that 2000 μg/mL of Scarb1-ASOs would also increase the G-CSF and CXCL1 expression in primary lung cells *in vitro*. Thus, these cells were treated with 8 to 2000 μg/mL of Scarb1-ASOs for 24 h to evaluate the immune-stimulatory effect. Unexpectedly, none of the doses of Scarb1-ASOs increased the mRNA or protein levels of G-CSF and CXCL1 (Fig 7A–7D), however, Scarb1-ASOs exerted a robust KD effect on primary lung cells *in vitro* (Fig 6). ApoB-ASOs did not increase the mRNA or protein levels of G-CSF and CXCL1 either (S3A–S3D Fig). In this *in vitro* experiment, ApoB-ASOs did not decrease the Scarb1 mRNA expression (S3E Fig). This result indicates that LNA-ASOs did not globally repress the gene expression. Although macrophages and lung epithelial cells existed in our *in vitro* assay system, and R848 significantly increased CXCL1 and G-CSF, LNA-ASOs did not induce an inflammatory response as observed in our *in vivo* experiment. This is because our *in vitro* assay system failed to mimic the *in vivo* environment perfectly. Further experiments are thus required to determine why LNA-ASOs did not induce an immune-stimulatory effect *in vitro*. To develop safe inhalation therapies using LNA-ASOs, we should prepare LNA-ASOs that will not induce abnormal immune responses. Our results suggest that *in vitro* assay systems using alveolar macrophages and lung epithelial cells cannot adequately evaluate the immune-stimulatory effect of LNA-ASOs. Therefore, appropriate *in vitro* assay systems are required to obtain safe LNA-ASOs, or else *in vivo* screening should be conducted.

Pre-clinical experiments with a focus on inhalation therapy with ASOs have been reported [40–46]. Some of these reports have shown that high doses of ASOs induced macrophage-associated inflammatory responses in the lung [47, 48]. However, no report has investigated both the *in vitro* and *in vivo* immune-stimulatory effect of ASOs, and the details of the mechanisms have yet to be clarified. Recently, two reports investigating the mechanism underlying the hepatotoxic side effects of LNA-ASOs were published [49, 50]. These reports indicated that hepatotoxicity of LNA-ASOs is caused by RNAseH1 activity, presumably because of off-target cleavage of RNAs inside nuclei. This mechanism may be associated with the inflammatory response in the lung induced by LNA-ASOs. However, in fact, LNA-ASOs did not induce inflammatory response in our *in vitro* experiment. Therefore, to investigate this hypothesis, *in vivo* experiments should be conducted rather than simple *in vitro* assays using primary lung cells.

In conclusion, we confirmed that the intratracheal administration of Scarb1-ASOs exerted both the KD of gene expression and a neutrophilic immune response in the murine lung *in vivo*. The dose required for KD in the lung was the same as the dose that induced a neutrophilic immune response. In addition, we could not estimate the *in vivo* immune-stimulatory effect of the LNA-ASOs by an *in vitro* assay using primary murine lung cells. In the present study, LNA-ASOs showed the potential to be used as an inhaled drug. However, our results strongly suggest that further study on the immune-stimulatory effects of LNA-ASOs in the lung will be necessary for the development of safe and effective inhaled LNA-ASO therapies.

## Supporting information

S1 Fig The effect of ApoB-ASOs on the G-CSF and Cxcl1 mRNA expression in the whole lung. ApoB-ASOs were intratracheally administered to C57BL/6 mice once a day, for 2 days. One day after the final administration, the right lung was collected and the G-CSF (A) and Cxcl1 mRNA (B) expression was measured.
(TIF)

S2 Fig The effect of ApoB-ASOs on the G-CSF and CXCL1 protein expression in the lung. ApoB-ASOs were intratracheally administered to C57BL/6 mice once a day, for 2 days. One day after the final administration, BALF was collected and the G-CSF (A) and CXCL1 protein (B) levels were measured. *, P < 0.05 (Wilcoxon rank sum test).
(TIF)

S3 Fig The effects of ApoB-ASOs on the G-CSF, CXCL1 and Scarb1 expression of murine primary lung cells. Primary lung cells were treated with the indicated concentrations of ApoB-ASOs for 24 h. After the treatment, the G-CSF (A) and CXCL1 (B) and Scarb1 (E) mRNA expression in primary lung cells, and the expression of G-CSF (C) and CXCL1 (D) protein in supernatant were measured. The values represent the mean ± SD of triplicate experiments. ***, P < 0.001, *, P < 0.05 versus the Medium group (Aspin-Welch test).
(TIF)

S1 File ARRIVE guidelines checklist. (PDF)

## References

[1] Bennett CF, Swayze EE (2010) RNA targeting therapeutics: molecular mechanisms of antisense oligonucleotides as a therapeutic platform. Annu Rev Pharmacol Toxicol 50: 259–93. doi: 10.1146/annurev.pharmtox.010909.105654 20055705 10.1146/annurev.pharmtox.010909.105654

[2] Kurreck J, Wyszko E, Gillen C, Erdmann VA (2002) Design of antisense oligonucleotides stabilized by locked nucleic acids. Nucleic Acids Res 30: 1911–1918. 11972327 10.1093/nar/30.9.1911 PMC113840

[3] Kauppinen S, Vester B, Wengel J (2005) Locked nucleic acid (LNA): High affinity targeting of RNA for diagnostics and therapeutics. Drug Discov Today Technol 2: 287–290. doi: 10.1016/j.ddtec.2005.08.012 24981949 10.1016/j.ddtec.2005.08.012 PMC7105916

[4] Veedu RN, Wengel J (2010) Locked nucleic acids: promising nucleic acid analogs for therapeutic applications. Chem Biodivers 7: 536–542. doi: 10.1002/cbdv.200900343 20232325 10.1002/cbdv.200900343

[5] Stein CA, Hansen JB, Lai J, Wu S, Voskresenskiy A, Anja H, et al (2010) Efficient gene silencing by delivery of locked nucleic acid antisense oligonucleotides, unassisted by transfection reagents. Nucleic Acids Res 38: e3 doi: 10.1093/nar/gkp841 19854938 10.1093/nar/gkp841 PMC2800216

[6] Soifer HS, Koch T, Lai J, Hansen B, Hoeg A, Oerum H, et al (2012) Silencing of gene expression by gymnotic delivery of antisense oligonucleotides. Methods Mol Biol 815: 333–346. doi: 10.1007/978-1-61779-424-7_25 22131003 10.1007/978-1-61779-424-7_25

[7] Zhang Y, Qu Z, Kim S, Shi V, Liao B, Kraft P et al (2011) Down-modulation of cancer targets using locked nucleic acid (LNA)-based antisense oligonucleotides without transfection. Gene Ther 18: 326–333. doi: 10.1038/gt.2010.133 Epub 2010 Dec 23. 21179173 10.1038/gt.2010.133 PMC3154478

[8] Straarup EM, Fisker N, Hedtjärn M, Lindholm MW, Rosenbohm C, Aarup V, et al (2010) Short locked nucleic acid antisense oligonucleotides potently reduce apolipoprotein B mRNA and serum cholesterol in mice and non-human primates. Nucleic Acids Res 38: 7100–7111. doi: 10.1093/nar/gkq457 Epub 2010 Jul 8. 20615897 10.1093/nar/gkq457 PMC2978335

[9] Gupta N, Fisker N, Asselin MC, Lindholm M, Rosenbohm C, Ørum H, et al (2010) A locked nucleic acid antisense oligonucleotide (LNA) silences PCSK9 and enhances LDLR expression in vitro and in vivo. PLoS One 17: e10682 doi: 10.1371/journal.pone.0010682 20498851 10.1371/journal.pone.0010682 PMC2871785

[10] Swayze EE, Siwkowski AM, Wancewicz EV, Migawa MT, Wyrzykiewicz TK, Hung G, et al (2007) Antisense oligonucleotides containing locked nucleic acid improve potency but cause significant hepatotoxicity in animals. Nucleic Acids Res 35: 687–700. doi: 10.1093/nar/gkl1071 17182632 10.1093/nar/gkl1071 PMC1802611

[11] Morihara H, Yamamoto T, Oiwa H, Tonegawa K, Tsuchiyama D, Kawakatsu I, et al (2016) Phospholamban Inhibition by a Single Dose of Locked Nucleic Acid Antisense Oligonucleotide Improves Cardiac Contractility in Pressure Overload-Induced Systolic Dysfunction in Mice. J Cardiovasc Pharmacol Ther. doi: 10.1177/1074248416676392 27811197 10.1177/1074248416676392

[12] Delgado E, Okabe H, Preziosi M, Russell JO, Alvarado TF, Oertel M, et al (2015) Complete response of Ctnnb1-mutated tumours to β-catenin suppression by locked nucleic acid antisense in a mouse hepatocarcinogenesis model. J Hepatol 62: 380–387. doi: 10.1016/j.jhep.2014.10.021 25457204 10.1016/j.jhep.2014.10.021 PMC4300253

[13] Frazier KS (2015) Antisense oligonucleotide therapies: the promise and the challenges from a toxicologic pathologist's perspective. Toxicol Pathol 43: 78–89. doi: 10.1177/0192623314551840 25385330 10.1177/0192623314551840

[14] Hizawa N (2015) LAMA/LABA vs ICS/LABA in the treatment of COPD in Japan based on the disease phenotypes. Int J Chron Obstruct Pulmon Dis 10: 1093–1102. doi: 10.2147/COPD.S72858 eCollection 2015. 26089659 10.2147/COPD.S72858 PMC4468951

[15] Wolthers OD (2016) Budesonide + formoterol fumarate dihydrate for the treatment of asthma. Expert Opin Pharmacother 17: 1023–1030. doi: 10.1517/14656566.2016.1165207 27070946 10.1517/14656566.2016.1165207

[16] Ikematsu H, Kawai N, Iwaki N, Kashiwagi S (2016) Duration of fever and other symptoms after the inhalation of laninamivir octanoate hydrate for influenza treatment; comparison among the four Japanese influenza seasons from 2011–2012 to 2014–2015. J Infect Chemother 22: 605–610. doi: 10.1016/j.jiac.2016.06.003 27493024 10.1016/j.jiac.2016.06.003

[17] Greenberg J, Palmer JB, Chan WW, Correia CE, Whalley D, Shannon P, et al (2016) Treatment satisfaction in cystic fibrosis: early patient experience with tobramycin inhalation powder. Patient Prefer Adherence 10: 2163–2169. doi: 10.2147/PPA.S102234 27822017 10.2147/PPA.S102234 PMC5087789

[18] Tanaka M, Nyce JW (2001) Respirable antisense oligonucleotides: a new drug class for respiratory disease. Respir Res 2: 5–9. doi: 10.1186/rr32 11686859 10.1186/rr32 PMC59563

[19] Crosby JR, Guha M, Tung D, Miller DA, Bender B (2007) Inhaled CD86 antisense oligonucleotide suppresses pulmonary inflammation and airway hyper-responsiveness in allergic mice. J Pharmacol Exp Ther 321: 938–946. doi: 10.1124/jpet.106.119214 17389243 10.1124/jpet.106.119214

[20] Lafferty EI, Qureshi ST, Schnare M (2010) The role of toll-like receptors in acute and chronic lung inflammation. J Inflamm (Lond) 7:57 doi: 10.1186/1476-9255-7-57 21108806 10.1186/1476-9255-7-57 PMC3003652

[21] Opitz B, van Laak V, Eitel J, Suttorp N (2010) Innate immune recognition in infectious and noninfectious diseases of the lung. Am J Respir Crit Care Med 181: 1294–1309. doi: 10.1164/rccm.200909-1427SO 20167850 10.1164/rccm.200909-1427SO

[22] Hussell T, Bell TJ (2014) Alveolar macrophages: plasticity in a tissue-specific context. Nat Rev Immunol 14: 81–93. doi: 10.1038/nri3600 24445666 10.1038/nri3600

[23] Kopf M, Schneider C, Nobs SP (2015) The development and function of lung-resident macrophages and dendritic cells. Nat Immunol 16: 36–44. doi: 10.1038/ni.3052 25521683 10.1038/ni.3052

[24] Eisele NA, Anderson DM (2011) Host Defense and the Airway Epithelium: Frontline Responses That Protect against Bacterial Invasion and Pneumonia. J Pathog doi: 10.4061/2011/249802 22567325 10.4061/2011/249802 PMC3335569

[25] Nishina K, Piao W, Yoshida-Tanaka K, Sujino Y, Nishina T, Yamamoto T, et al (2015) DNA/RNA heteroduplex oligonucleotide for highly efficient gene silencing. Nat Commun 10;6:7969 doi: 10.1038/ncomms8969 26258894 10.1038/ncomms8969 PMC4918363

[26] Shen WJ, Hu J, Hu Z, Kraemer FB, Azhar S (2014) Scavenger receptor class B type I (SR-BI): a versatile receptor with multiple functions and actions. Metabolism 63(7):875–886. doi: 10.1016/j.metabol.2014.03.011 24854385 10.1016/j.metabol.2014.03.011 PMC8078058

[27] Murray S, Ittig D, Koller E, Berdeja A, Chappell A, Prakash TP, et al (2012) TricycloDNA-modified oligo-2'-deoxyribonucleotides reduce scavenger receptor B1 mRNA in hepatic and extra-hepatic tissues—a comparative study of oligonucleotide length, design and chemistry. Nucleic Acids Res 40: 6135–6143. doi: 10.1093/nar/gks273 22467214 10.1093/nar/gks273 PMC3401458

[28] Zimmermann TS, Lee AC, Akinc A, Bramlage B, Bumcrot D, Fedoruk MN, et al (2006) RNAi-mediated gene silencing in non-human primates. Nature 441(7089):111–114. doi: 10.1038/nature04688 16565705 10.1038/nature04688

[29] Guo L, Song Z, Li M, Wu Q, Wang D, Feng H, et al (2009) Scavenger Receptor BI Protects against Septic Death through Its Role in Modulating Inflammatory Response. J Biol Chem 284(30):19826–34. doi: 10.1074/jbc.M109.020933 19491399 10.1074/jbc.M109.020933 PMC2740408

[30] Feng H, Guo L, Wang D, Gao H, Hou G, Zheng Z, et al Deficiency of scavenger receptor BI leads to impaired lymphocyte homeostasis and autoimmune disorders in mice. Arterioscler Thromb Vasc Biol 31(11):2543–51. doi: 10.1161/ATVBAHA.111.234716 21836069 10.1161/ATVBAHA.111.234716 PMC3202973

[31] Bivas-Benita M, Zwier R, Junginger HE, Borchard G (2005) Non-invasive pulmonary aerosol delivery in mice by the endotracheal route. Eur J Pharm Biopharm. 61(3):214–8. doi: 10.1016/j.ejpb.2005.04.009 16039104 10.1016/j.ejpb.2005.04.009

[32] van Rijt LS, Kuipers H, Vos N, Hijdra D, Hoogsteden HC, Lambrecht BN (2004) A rapid flow cytometric method for determining the cellular composition of bronchoalveolar lavage fluid cells in mouse models of asthma. J Immunol Methods 288: 111–121. doi: 10.1016/j.jim.2004.03.004 15183090 10.1016/j.jim.2004.03.004

[33] Chua F, Dunsmore SE, Clingen PH, Mutsaers SE, Shapiro SD, Segal AW, et al (2004) Mice lacking neutrophil elastase are resistant to bleomycin-induced pulmonary fibrosis. Am J Pathol 170: 65–74. doi: 10.2353/ajpath.2007.060352 17200183 10.2353/ajpath.2007.060352 PMC1762691

[34] Eyles JL, Hickey MJ, Norman MU, Croker BA, Roberts AW, Drake SF, et al (2008) A key role for G-CSF-induced neutrophil production and trafficking during inflammatory arthritis. Blood 112: 5193–5201. doi: 10.1182/blood-2008-02-139535 18824600 10.1182/blood-2008-02-139535

[35] Singh P, Hu P, Hoggatt J, Moh A, Pelus LM (2012) Expansion of bone marrow neutrophils following G-CSF administration in mice results in osteolineage cell apoptosis and mobilization of hematopoietic stem and progenitor cells. Leukemia 26: 2375–2383. doi: 10.1038/leu.2012.117 22543963 10.1038/leu.2012.117 PMC3410045

[36] De Filippo K, Dudeck A, Hasenberg M, Nye E, van Rooijen N, Hartmann K, et al (2013) Mast cell and macrophage chemokines CXCL1/CXCL2 control the early stage of neutrophil recruitment during tissue inflammation. Blood 121: 4930–4937. doi: 10.1182/blood-2013-02-486217 23645836 10.1182/blood-2013-02-486217

[37] Braber S, Overbeek SA, Koelink PJ, Henricks PA, Zaman GJ, Garssen J, et al (2011) CXCR2 antagonists block the N-Ac-PGP-induced neutrophil influx in the airways of mice, but not the production of the chemokine CXCL1. Eur J Pharmacol 668: 443–449. doi: 10.1016/j.ejphar.2011.03.025 21458445 10.1016/j.ejphar.2011.03.025

[38] van Haren SD, Dowling DJ, Foppen W, Christensen D, Andersen P, Reed SG, et al (2016) Age-Specific Adjuvant Synergy: Dual TLR7/8 and Mincle Activation of Human Newborn Dendritic Cells Enables Th1 Polarization. J Immunol 197: 4413–4424. doi: 10.4049/jimmunol.1600282 27793997 10.4049/jimmunol.1600282 PMC7386828

[39] Duechs MJ, Hahn C, Benediktus E, Werner-Klein M, Braun A, Hoymann HG, et al (2011) TLR agonist mediated suppression of allergic responses is associated with increased innate inflammation in the airways. Pulm Pharmacol Ther 24: 203–214. doi: 10.1016/j.pupt.2010.12.009 21195789 10.1016/j.pupt.2010.12.009

[40] Stenton GR, Kim MK, Nohara O, Chen CF, Hirji N, Wills FL, et al (2000) Aerosolized Syk antisense suppresses Syk expression, mediator release from macrophages, and pulmonary inflammation. J Immunol. 4 1;164(7):3790–3797. Erratum in: J Immunol. 15;164(10):5532. 10725739 10.4049/jimmunol.164.7.3790

[41] Stenton GR, Ulanova M, Déry RE, Merani S, Kim MK, Gilchrist M, et al Inhibition of allergic inflammation in the airways using aerosolized antisense to Syk kinase. J Immunol. 15;169(2):1028–1036. 12097411 10.4049/jimmunol.169.2.1028

[42] Duan W, Chan JH, McKay K, Crosby JR, Choo HH, Leung BP, et al (2005) Inhaled p38alpha mitogen-activated protein kinase antisense oligonucleotide attenuates asthma in mice. Am J Respir Crit Care Med. 15;171(6):571–578. doi: 10.1164/rccm.200408-1006OC 15557129 10.1164/rccm.200408-1006OC

[43] Karras JG, Crosby JR, Guha M, Tung D, Miller DA, Gaarde WA, et al (2007) Anti-inflammatory activity of inhaled IL-4 receptor-alpha antisense oligonucleotide in mice. Am J Respir Cell Mol Biol. 36(3):276–285. doi: 10.1165/rcmb.2005-0456OC 16990616 10.1165/rcmb.2005-0456OC

[44] Crosby JR1, Guha M, Tung D, Miller DA, Bender B, Condon TP, et al (2007) Inhaled CD86 antisense oligonucleotide suppresses pulmonary inflammation and airway hyper-responsiveness in allergic mice. J Pharmacol Exp Ther. 321(3):938–946. doi: 10.1124/jpet.106.119214 17389243 10.1124/jpet.106.119214

[45] Allakhverdi Z, Allam M, Renzi PM. (2006) Inhibition of antigen-induced eosinophilia and airway hyperresponsiveness by antisense oligonucleotides directed against the common beta chain of IL-3, IL-5, GM-CSF receptors in a rat model of allergic asthma. Am J Respir Crit Care Med. 1;165(7):1015–1021. doi: 10.1164/ajrccm.165.7.2109095 11934731 10.1164/ajrccm.165.7.2109095

[46] Ripple MJ, You D, Honnegowda S, Giaimo JD, Sewell AB, Becnel DM, et al (2010) Immunomodulation with IL-4R alpha antisense oligonucleotide prevents respiratory syncytial virus-mediated pulmonary disease. J Immunol. 15;185(8):4804–4811. doi: 10.4049/jimmunol.1000484 20861354 10.4049/jimmunol.1000484 PMC3063095

[47] Guimond A, Viau E, Aubé P, Renzi PM, Paquet L, Ferrari N. (2008) Advantageous toxicity profile of inhaled antisense oligonucleotides following chronic dosing in non-human primates. Pulm Pharmacol Ther. 21(6):845–854. doi: 10.1016/j.pupt.2008.08.001 18761414 10.1016/j.pupt.2008.08.001

[48] Alton EW1, Boushey HA, Garn H, Green FH, Hodges M, Martin RJ, et al (2012) Clinical expert panel on monitoring potential lung toxicity of inhaled oligonucleotides: consensus points and recommendations. Nucleic Acid Ther. 22(4):246–254. doi: 10.1089/nat.2012.0345 22809313 10.1089/nat.2012.0345 PMC3426204

[49] Burel SA, Hart CE, Cauntay P, Hsiao J, Machemer T, Katz M, et al (2016) Hepatotoxicity of high affinity gapmer antisense oligonucleotides is mediated by RNase H1 dependent promiscuous reduction of very long pre-mRNA transcripts. Nucleic Acids Res. 18;44(5):2093–109. doi: 10.1093/nar/gkv1210 26553810 10.1093/nar/gkv1210 PMC4797265

[50] Kasuya T, Hori S, Watanabe A, Nakajima M, Gahara Y, Rokushima M, et al (2016) Ribonuclease H1-dependent hepatotoxicity caused by locked nucleic acid-modified gapmer antisense oligonucleotides. Sci Rep. 27;6:30377 doi: 10.1038/srep30377 27461380 10.1038/srep30377 PMC4961955

