# Supplementary figures and images for: The intratracheal administration of locked nucleic acid containing antisense oligonucleotides induced gene silencing and an immune-stimulatory effect in the murine lung
Source: PLoS One. 2017 Nov 6;12(11):e0187286. doi: 10.1371/journal.pone.0187286 (PMC5673232; doi:10.1371/journal.pone.0187286)

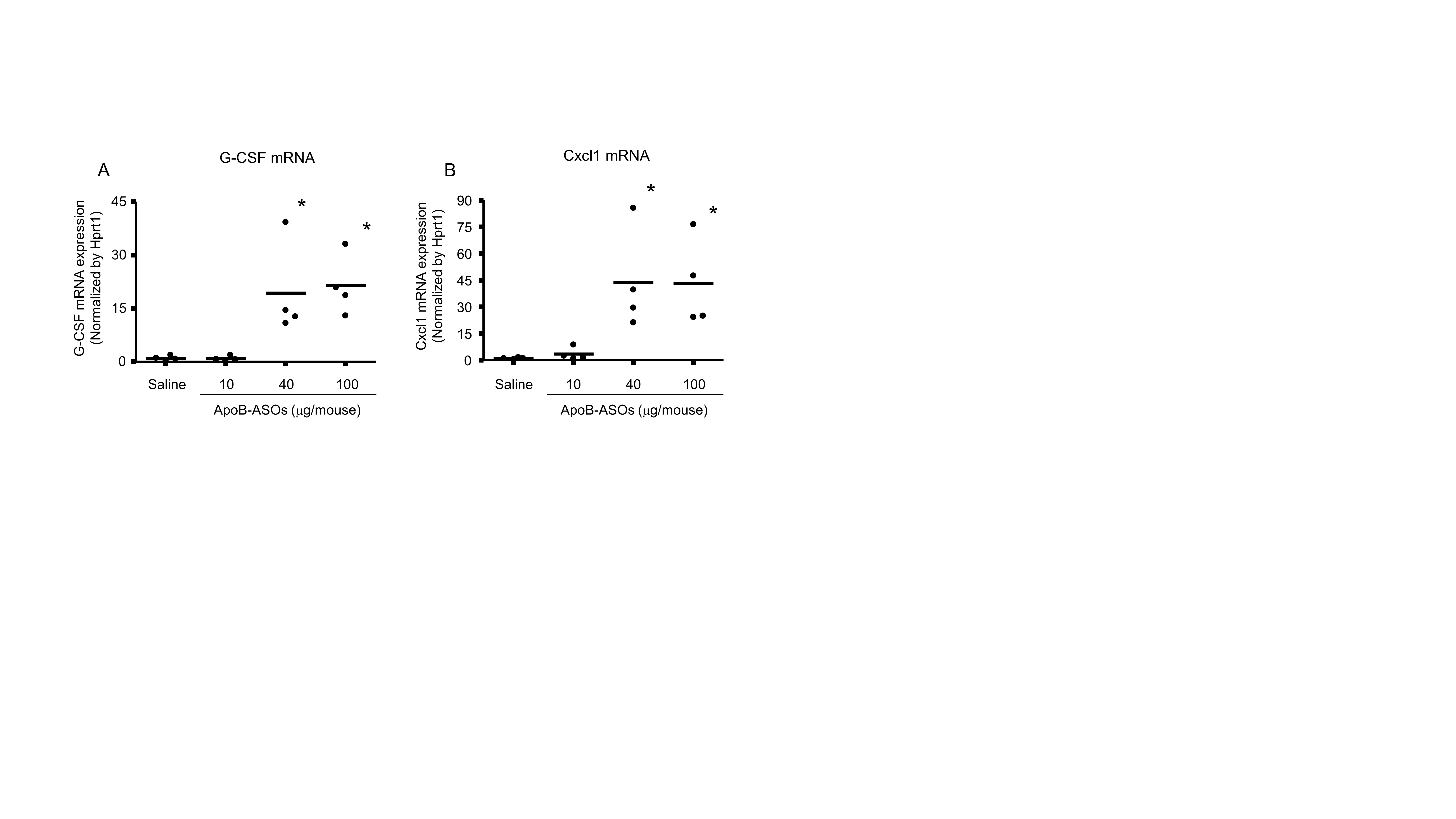

Supplement: S1 Fig — ApoB-ASOs were intratracheally administered to C57BL/6 mice once a day, for 2 days. One day after the final administration, the right lung was collected and the G-CSF (A) and Cxcl1 mRNA (B) expression was measured. (TIF) [file pone.0187286.s001.TIF]

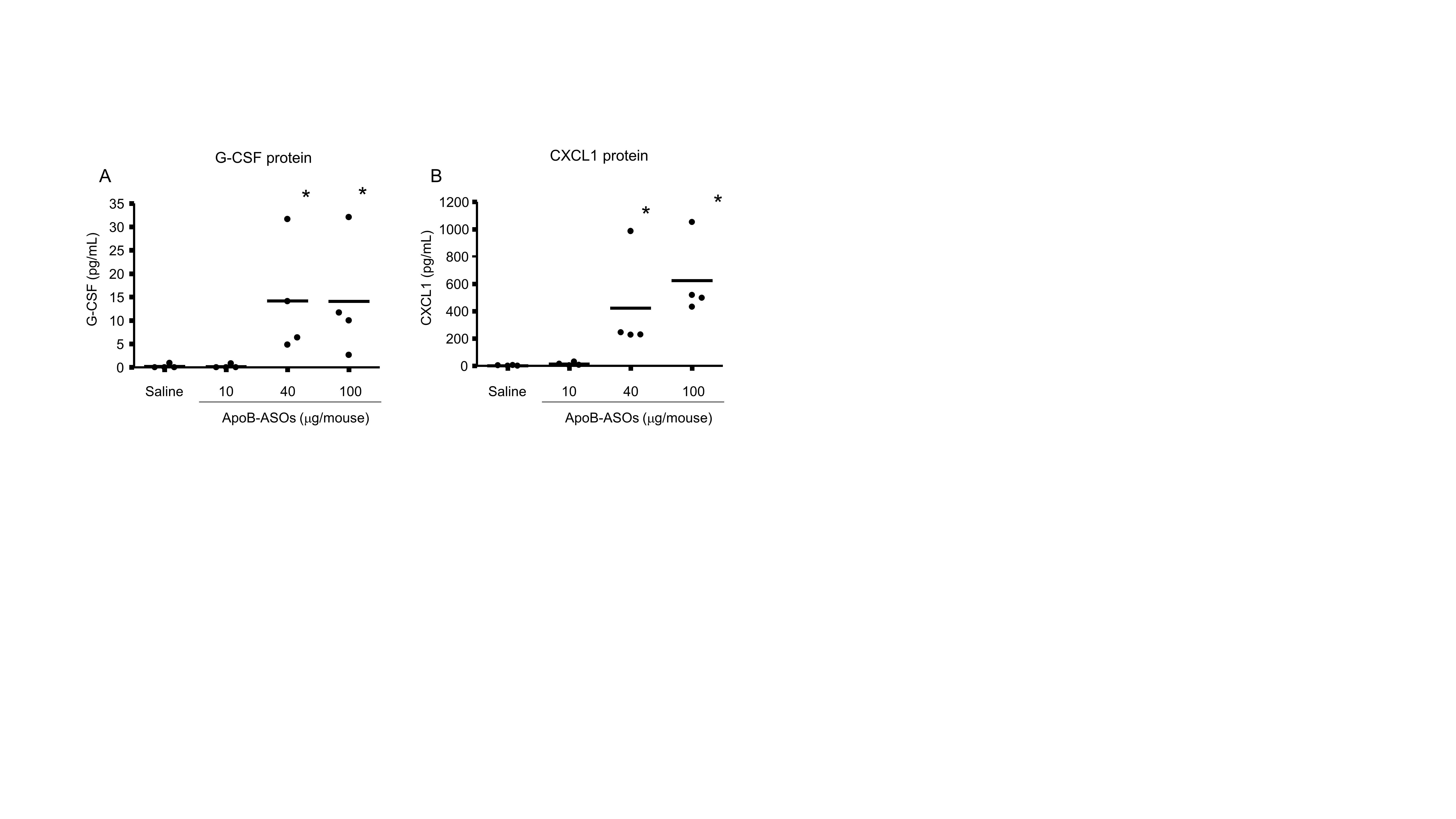

Supplement: S2 Fig — ApoB-ASOs were intratracheally administered to C57BL/6 mice once a day, for 2 days. One day after the final administration, BALF was collected and the G-CSF (A) and CXCL1 protein (B) levels were measured. *, P < 0.05 (Wilcoxon rank sum test). (TIF) [file pone.0187286.s002.TIF]

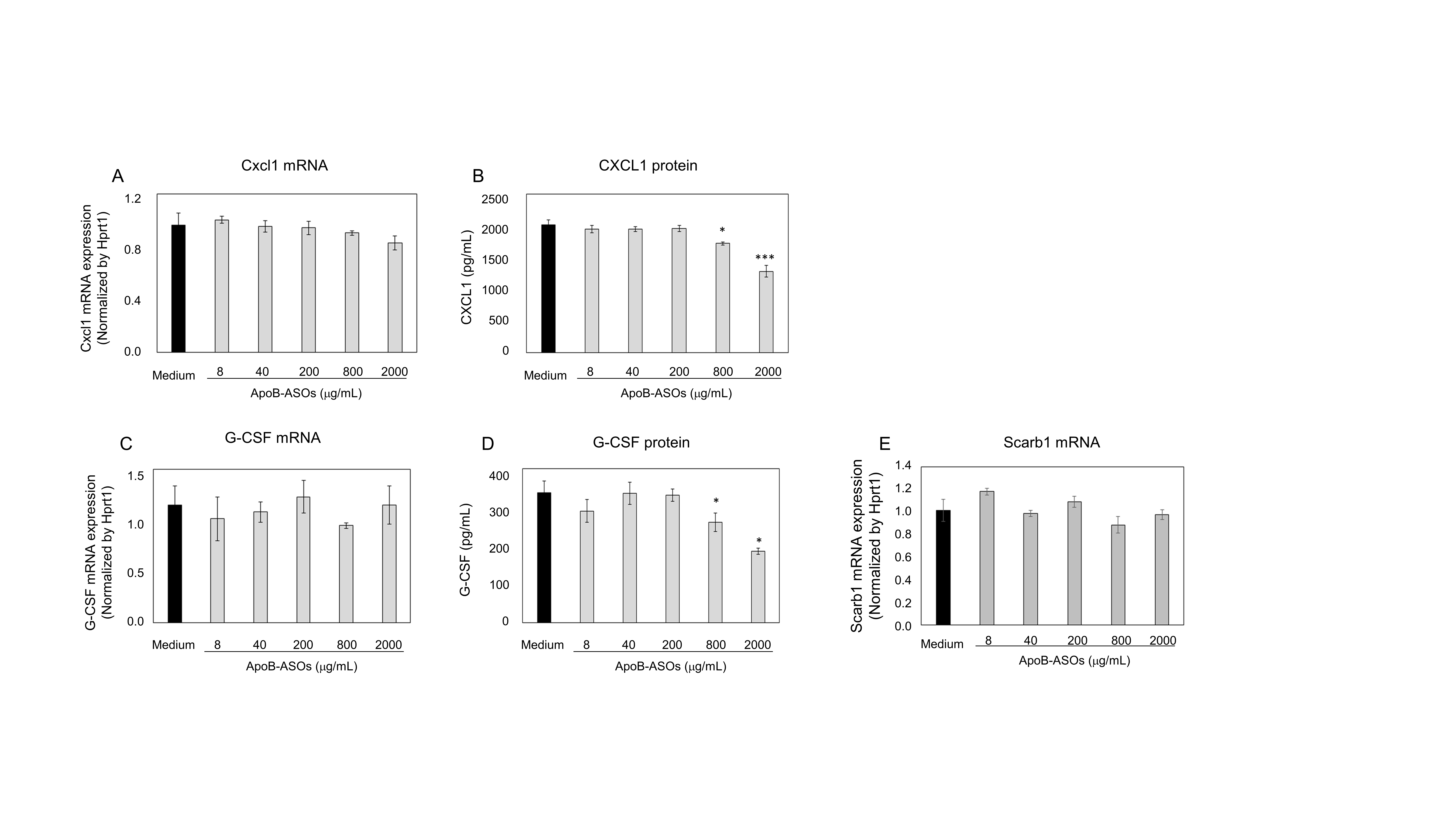

Supplement: S3 Fig — Primary lung cells were treated with the indicated concentrations of ApoB-ASOs for 24 h. After the treatment, the G-CSF (A) and CXCL1 (B) and Scarb1 (E) mRNA expression in primary lung cells, and the expression of G-CSF (C) and CXCL1 (D) protein in supernatant were measured. The values represent the mean ± SD of triplicate experiments. ***, P < 0.001, *, P < 0.05 versus the Medium group (Aspin-Welch test). (TIF) [file pone.0187286.s003.tif]
